# Editorial: New advances on hypothalamus-pituitary stem cell biology

**DOI:** 10.3389/fendo.2024.1392076

**Published:** 2024-03-11

**Authors:** Leonard Cheung, Shannon W. Davis, Maria Ines Perez-Millan

**Affiliations:** ^1^ Department of Physiology & Biophysics, Stony Brook University, Stony Brook, NY, United States; ^2^ Department of Biological Sciences, University of South Carolina, Columbia, SC, United States; ^3^ Instituto de Biociencias, Biotecnología y Biología Traslacional (iB3), Facultad de Ciencias Exactas y Naturales, Universidad de Buenos Aires, Buenos Aires, Argentina

**Keywords:** pituitary, hypothalamus, stem cell, genetics, organoids

The discovery of stem cells in many tissues including the pituitary and hypothalamus has presented the potential for stem cell regeneration and treatment for human diseases. However, significant gaps in knowledge remain in our understanding of the full range of molecular mechanisms that regulate the progression of these stem cells into the desired cell-types, limiting the translatability of basic science into human therapies. The articles in this Research Topic present novel data and review recent discoveries from a combination of human genetic studies, human organoid models and mouse models to improve our understanding of hypothalamic-pituitary stem cell regulation.

The median eminence of the hypothalamus is the critical interface between the brain and the pituitary gland. In addition to neurons, it contains multiple non-neural cell-types including oligodendrocyte precursors and stem cell-like β2-tanycytes. In Clayton et al . the authors discuss recent discoveries regarding these various cell-types and their regulatory mechanisms, including the role of diet on tanycytes, as well as future questions that remain as we continue to understand the central role of the median eminence in the neuroendocrine system.

Improvements in genomic sequencing techniques have continued to increase the number of genetic variants associated with hypothalamic-pituitary disease. Functional studies can subsequently demonstrate the mechanisms by which genes regulate stem cell differentiation into differentiated cells. In Martinez-Mayer and Perez-Millan the authors review the changing landscape of patients described with variants in the G-coupled receptor *PROKR2*, which were originally discovered to cause hypothalamic phenotypes in Kallman syndrome patients. More recently PROKR2 variants have been associated with pituitary disease, leading the authors to consider a direct role in regulating pituitary hormone cell specification. In Bando et al. the authors review recent cases describing novel genes associated with pituitary disease that require functional studies to determine the mechanisms that disrupt hormone production including possible involvement of pituitary stem cells. The authors also consider novel molecular mechanisms of variants in known pituitary disease genes such as synonymous variants that alter splicing of the combined pituitary hormone deficiency gene *POU1F1*.

A prominent aspect of stem cell therapies is the utilization of *in vitro* differentiated cells. Human embryonic stem cells (hESCs) and induced pluripotent stem cells (hiPSCs) have both been differentiated into hormone-producing pituitary organoids *in vitro* and successfully re-implanted into mice to rescue induced hypopituitarism. However, *in vitro* generation of pituitary organoids remains difficult and is limited by the minor proportion of organoids that differentiate into pituitary cells, reinforcing the need for further methodological refinements required before potential stem cell therapies. In Kodani et al. the authors performed cell surface protein profiling of pituitary organoids and identify expression of EpCAM as an anterior pituitary-specific cell surface marker, allowing for their enrichment and reaggregation into high-proportion pituitary organoids that respond well to hypothalamic neuropeptide hormones.

Another aspect of stem cell therapies considers how to activate endogenous pituitary stem cells to differentiate into particular hormone cell-types in order to regenerate damaged tissue. Pituitary stem cells have been shown to produce nascent hormone cells in response to induced cell death and pathological demand. In Laporte et al. the authors show that cytokines such as interleukin IL6 play an important role in activation of pituitary stem cell proliferation in response to diphtheria-induced tissue damage and pose the question of how cytokines may be modulated to aid or improve this process in the future.

In sum, the articles in this Research Topic present an update to the current understanding of hypothalamic and pituitary stem cells, and discuss future directions for investigation, potentially bringing us closer to the realization of effective and safe stem cell therapies to combat hypothalamic and pituitary diseases in humans.

## Author contributions

LC: Writing – review & editing, Writing – original draft. SD: Writing – review & editing, Writing – original draft. MM: Writing – review & editing, Writing – original draft.

